# Human-Induced Pluripotent Stem Cell–Derived Cardiomyocyte Model for *TNNT2* Δ160E-Induced Cardiomyopathy

**DOI:** 10.1161/CIRCGEN.121.003522

**Published:** 2022-07-12

**Authors:** Takumi Kondo, Shuichiro Higo, Mikio Shiba, Yasuaki Kohama, Satoshi Kameda, Tomoka Tabata, Hiroyuki Inoue, Shota Okuno, Shou Ogawa, Satoki Nakamura, Maki Takeda, Emiko Ito, Junjun Li, Li Liu, Yuki Kuramoto, Jong-Kook Lee, Seiji Takashima, Shigeru Miyagawa, Yoshiki Sawa, Shungo Hikoso, Yasushi Sakata

**Affiliations:** Department of Cardiovascular Medicine (T.K., M.S., S.K., T.T., H.I., S. Okuno, S.Ogawa, Y.K., J.-K.L., S. Hikoso, Y.S.); Department of Medical Therapeutics for Heart Failure (S. Higo); Department of Cardiovascular Surgery (M.T., E.I., J.L., L.L., S.M., Y.S.); Department of Cardiovascular Regenerative Medicine (J.-K.L.); Department of Medical Biochemistry (S.T.), Osaka University Graduate School of Medicine, Suita, Osaka.; National Hospital Organization, Osaka-Minami Medical Center, Kawachinagano, Osaka (Y.K.).; Osaka Police Hospital, Osaka (S.N.).

**Keywords:** cardiomyopathy, hypertrophic, heart failure, mutation, phenotype, prognosis, troponin

## Abstract

The Δ160E mutation in *TNNT2*, which encodes troponin T, is a rare pathogenic variant identified in patients with hypertrophic cardiomyopathy and is associated with poor prognosis. Thus, a convenient human model recapitulating the pathological phenotype caused by *TNNT2* Δ160E is required for therapeutic development.

**Methods:**

We identified a heterozygous in-frame deletion mutation (c.478_480del, p.Δ160E) in *TNNT2* in a patient with familial hypertrophic cardiomyopathy showing progressive left ventricular systolic dysfunction, leading to advanced heart failure. To investigate the pathological phenotype caused by Δ160E, we generated a set of isogenic induced pluripotent stem cells carrying the heterozygous Δ160E, homozygously corrected or homozygously introduced Δ160E using genome editing and differentiated them into cardiomyocytes (Hetero-Δ160E-, wild type-, and Homo-Δ160E-induced pluripotent stem cells [iPSC]-derived cardiomyocytes [iPSC-CMs]).

**Results:**

Hetero-Δ160E-iPSC-CMs exhibited prolonged calcium decay, relaxation impairment, and hypertrophy compared to wild type-iPSC-CMs. Notably, these phenotypes were further exacerbated in Homo-Δ160E-iPSC-CMs. Overexpression of R-GECO-fused Δ160E mutant troponin T prolonged decay time and time to peak of the myofilament-localized calcium transient in iPSC-CMs, indicating that sarcomeric calcium retention with Δ160E may affect intracellular calcium concentration. High-content imaging analysis detected remarkable nuclear translocation of NFATc1, especially in Homo-Δ160E-iPSC-CMs, indicating that the Δ160E mutation promotes hypertrophic signaling pathway in a dose-dependent manner. Increased phosphorylation of CaMKIIδ (calcium/calmodulin-dependent protein kinase IIδ) and phospholamban at Thr17 was observed in Homo- and Hetero-Δ160E-iPSC-CMs. Epigallocatechin-3-gallate, a calcium desensitizing compound, shortened prolonged calcium decay and relaxation duration in Δ160E-iPSC-CMs.

**Conclusions:**

Isogenic iPSC-CMs recapitulate the prolonged calcium decay, relaxation impairment, and subsequent calcium-regulated signaling pathways caused by the *TNNT2* Δ160E mutation and can serve as a human model for therapeutic development to prevent hypertrophic cardiomyopathy pathology.

Hypertrophic cardiomyopathy (HCM) is one of the most common inherited cardiovascular disease, with a prevalence of ≈ 1 in 500 people.^1^ HCM is inherited with an autosomal dominant Mendelian pattern and almost half of the patients with familial HCM possess pathogenic variants of genes encoding cardiac sarcomeric proteins.^1^ Although ≈ 80% of genotype-positive patients with HCM harbor pathogenic variants of thick filament genes, 20% of the patients possess variants of thin filament genes such as *TNNT2* (TnT [troponin T]), *TNNI3* (troponin I), *TPM1* (α-tropomyosin), and *ACTC* (cardiac actin).^2^ Cohort studies linking clinical and genetic information have demonstrated that patients with HCM harboring thin filament mutations including *TNNT2*, show increased risk of adverse remodeling, left ventricular systolic dysfunction^3,4^ and sudden death.^5^ Among *TNNT2* mutations, in-frame deletion of 3 nucleotides encoding a glutamic acid at position 160 of the protein (Δ160E) is a rare pathogenic variant that was first reported in familial HCM cases in 1995^6^; it is identified in 1 of 112 familial HCM cases in Japan,^7^ in 1 of 197 familial or sporadic HCM cases in France,^8^ and in 3 of 552 HCM cases in the United Kingdom,^9^ indicating its global prevalence.

The Δ160E mutation is located in the linker region of TnT and decreases flexibility of TNT1.^10^ Although it is difficult to identify the precise conformation of the linker region of TnT in crystal structures because of its flexible nature, atomistic models and biochemical analyses have revealed that Δ160E augments the calcium affinity of troponin C and increases calcium sensitivity.^11^ Extensive studies using transgenic mice expressing the Δ160E mutant TnT have demonstrated that this mutation increases calcium sensitivity and promotes energy wastage in tension generation and secondary calcium handling abnormalities.^12,13^ As the *TNNT2* Δ160E mutation has been identified worldwide and is associated with high risk of sudden cardiac death and poor clinical prognosis in patients with HCM,^6–9^ establishment of a convenient human disease model that recapitulates the pathological phenotype caused by *TNNT2* Δ160E mutation under an isogenic background is critical for appropriate therapeutic development.

Here, we recruited a patient with familial HCM who showed progressive left ventricular systolic dysfunction leading to advanced heart failure, carrying a heterozygous *TNNT2* Δ160E mutation; we then generated a set of isogenic induced pluripotent stem cell (iPSC) clones with 3 distinct genotypes using CRISPR/Cas9 genome editing, and examined their potential as a disease model for HCM.

## Materials and Methods

Information about detailed materials and methods in this study is available in Supplemental Methods. The authors declare that all supporting data are available within the article and its Supplemental Material. The use of human samples and the genomic analysis were approved by the Ethics Committee of Osaka University Hospital, and written informed consent was obtained from the participant. This investigation conformed to Ethical Guidelines for Medical and Health Research Involving Human Subjects in Japan and all principles outlined in the Declaration of Helsinki.

## Results

### Identification of a Heterozygous *TNNT2* Δ160E Mutation in a Patient With Familial HCM Showing Left Ventricular Systolic Dysfunction Leading to Advanced Heart Failure

**Figure 1.**
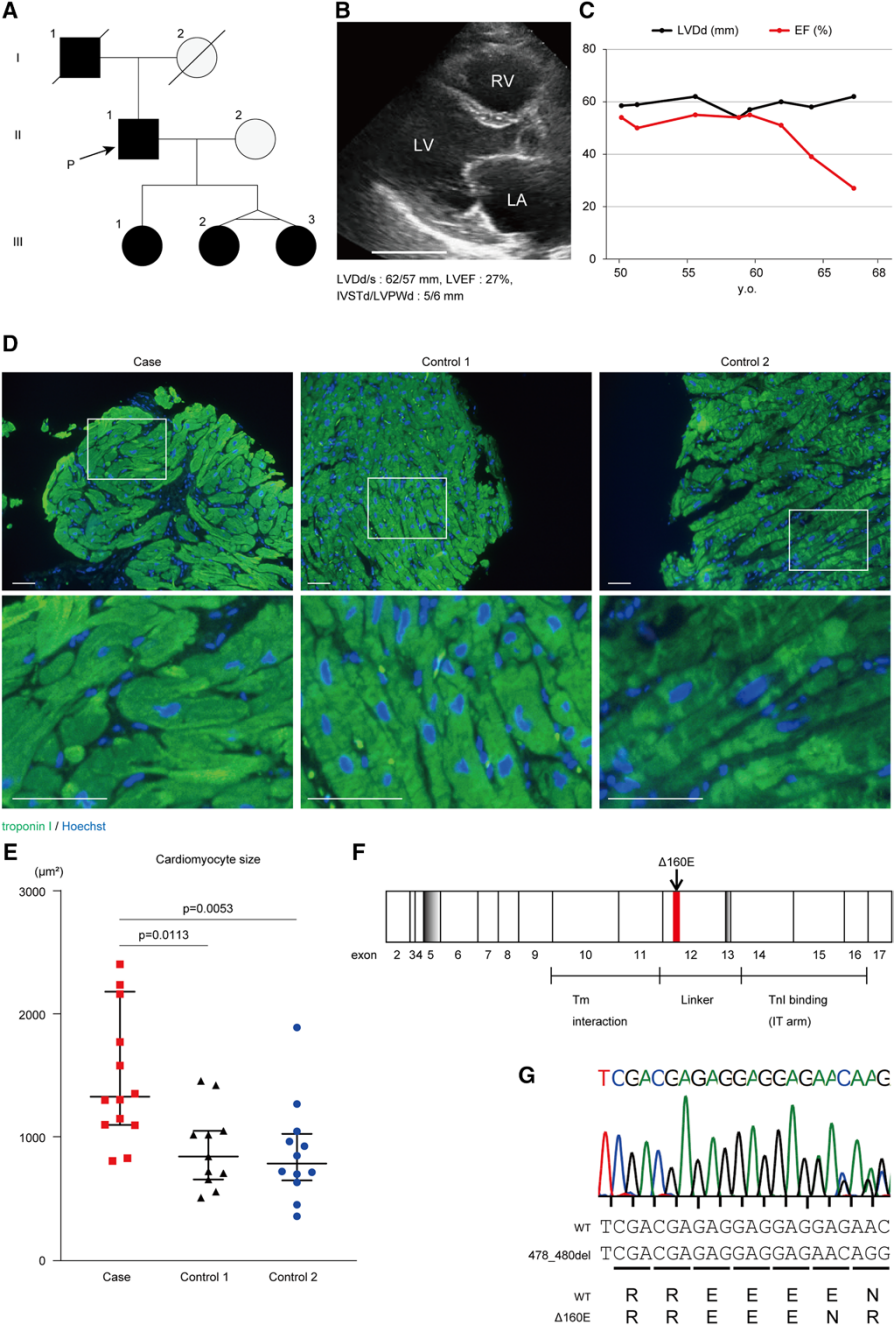
**Heterozygous Δ160E mutation in TNNT2 was identified in the patient with hypertrophic cardiomyopathy and progressive systolic dysfunction. A**, Family pedigree chart of the proband. The 5 patients who were diagnosed with hypertrophic cardiomyopathy are indicated by black circles (female) or black boxes (male). **B**, A parasternal left ventricle long-axis view of the echocardiogram of the proband at age 66 is shown. Scale bar: 50 mm. **C**, Time course of left ventricular diastolic diameter and ejection fraction in the echocardiogram of the proband. **D**, Immunofluorescence images of the myocardium obtained via endomyocardial biopsy of the right ventricle in the proband or control cases. Scale bar: 50 μm. **E**, Analysis of cardiomyocyte size in immunofluorescence images of the myocardium from the proband and other patients with cardiomyopathy (number of analyzed cells: the proband, 100; control 1, 205; control 2, 224). Data are shown as Dot plots for each group. **F**, Exon-organization of human cardiac troponin T. The regions thought to interact with tropomyosin (Tm) and troponin I (TnI) are illustrated. The Δ160E mutation is located at the proximal site of the linker lesion in exon 12. **G**, Direct Sanger sequence analysis of the *TNNT2* locus using genomic DNA obtained from the proband. EF indicates ejection fraction; IVSTd/LVPWd, diastolic interventricular septum thickness, and left ventricular posterior wall thickness; LA, left atrium; LV, left ventricle; LVDd/s, left ventricular diastolic and systolic diameter; and RV, right ventricle.

We recruited a 68-year-old male patient with HCM showing left ventricular systolic dysfunction. He had a family history of HCM in his father and 3 daughters (Figure 1A). He had been diagnosed with HCM at the age of 25 years. According to the available medical records, enlargement of the left ventricle was detected at age 50, and the left ventricular ejection fraction gradually decreased after age 60 (Figure 1B and 1C). He received cardiac-resynchronization therapy at the age of 65 years for primary prevention. He experienced sustained ventricular tachycardia at age 67 and died of heart failure at the age of 68 years. Immunofluorescent staining analysis of the patient’s myocardium revealed remarkable cardiomyocyte hypertrophy in the proband compared with that in age-matched patients with nonischemic cardiomyopathy and systolic dysfunction (Figure 1D and 1E, 1334 [1104–2187] μm^2^; *P*=0.0113 versus control 1, 848 [661–1057] μm^2^; *P*=0.0053 versus control 2, 790 [653–1031] μm^2^). In total, we screened a total of 404 genes related to inherited cardiovascular disease using the Ion Ampliseq cardiovascular research panel^14^ and confirmed a previously reported heterozygous in-frame 3-base pair deletion (c.478_480del, p.Δ160E) in the *TNNT2* gene in his genome and the genome of his 3 daughters (Figure 1F and 1G).

### Generation of Disease-Specific iPSCs and a Set of Isogenic iPSCs Using CRISPR/Cas9 Genome Editing and Their Differentiation to Cardiomyocytes

**Figure 2.**
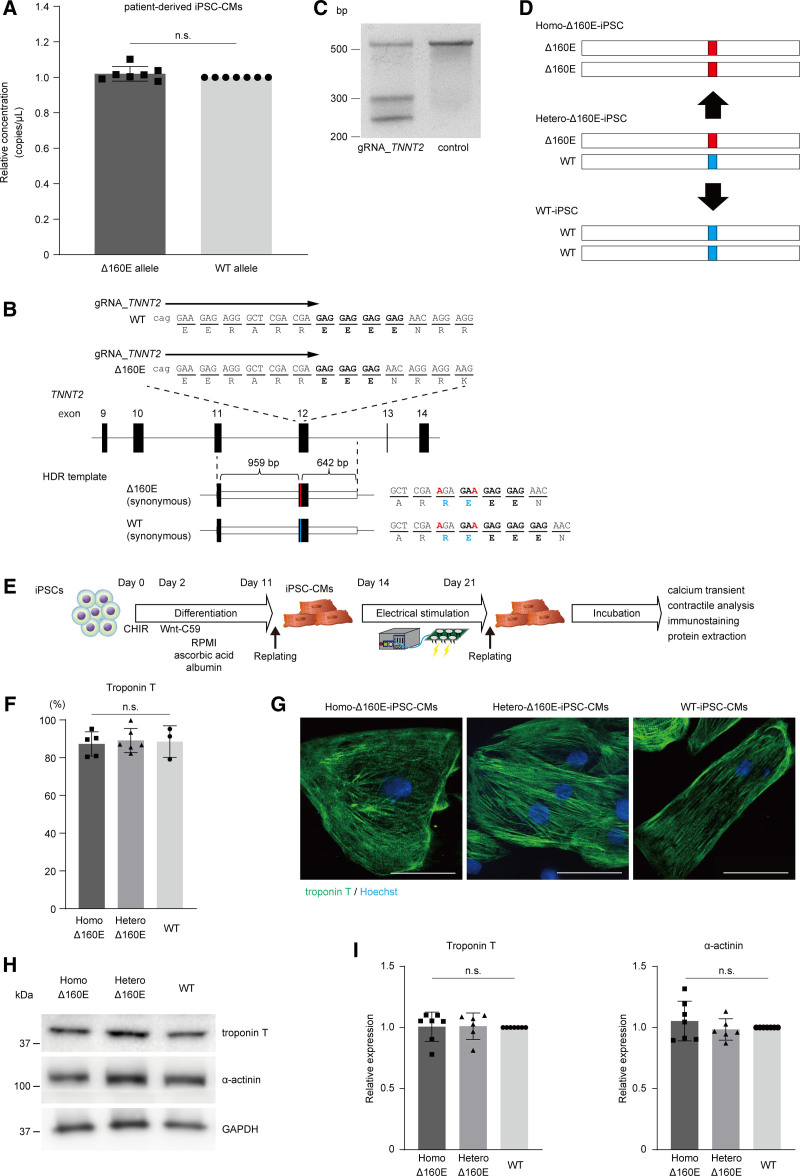
**A set of isogenic induced pluripotent stem cell -derived cardiomyocytes (iPSC-CMs) with corrected wild type (WT), heterozygous Δ160E and introduced homozygous Δ160E TNNT2 mutation were generated using genome editing. A**, Results of droplet digital polymerase chain reaction ([PCR]; ddPCR) analysis using cDNA samples obtained from patient-derived iPSC-CMs (n=7, biological replicates). The concentration (copies/μL) of each *TNNT2* transcript in the cDNA samples was calculated from ddPCR analysis using Poisson distribution. The concentration of transcripts from the Δ160E allele relative to the WT allele in the cDNA of patient-derived iPSC-CMs is shown. **B**, The targeted site of genome editing around the Δ160E mutation in exon 12 of the human *TNNT2* gene. gRNA_*TNNT2* targets just upstream of the repeated GAG sequences. Design of HDR templates with Δ160E and WT sequences consisting of the 959-bp 5´-terminal and 642-bp 3´-terminal homologous arms corresponding to the genomic sequence around exon 12 of *TNNT2* are shown. Synonymous mutations were introduced to avoid Cas9-mediated re-cleavage. **C**, Cleaving activities of gRNA_*TNNT2* targeting the endogenous *TNNT2* locus in HEK293T cells were evaluated using the Cel-I assay **D**, Scheme for the generation of isogenic iPSCs. The Δ160E mutation was corrected to the normal allele (wild type [WT]) or homozygously introduced (Homo-Δ160E). **E**, Time course of monolayered differentiation into cardiomyocytes. Differentiated cardiomyocytes were replated, electrically stimulated at 2 Hz for 7 days, and then replated for further analysis. **F**, The proportion of TnT-positive cells in the isogenic iPSC-CM set was determined by flow cytometry (n=6, mean±SD). **G**, The set of isogenic iPSC-CMs replated on chamber slides was fixed at 3 wk after differentiation and immunostained with the indicated antibodies. Nuclei were detected using Hoechst staining. Scale bar: 50 μm. **H**, Whole-cell lysates were extracted from Homo-Δ160E-, Hetero-Δ160E-, and WT-iPSC-CMs at 4 to 5 wk after differentiation and were analyzed by Western blotting using the indicated antibodies. **I**, Quantified protein expression levels normalized to GAPDH expression were compared among the 3 groups (n=7, mean±SD).

We generated iPSCs from peripheral blood mononuclear leukocytes obtained from the proband. The generated iPSCs were positive for SSEA4, TRA-1-60, OCT3/4, and NANOG, and possessed a normal karyotype (Figure S1A and S1B). We differentiated them into cardiomyocytes using a chemically defined protocol, as described previously.^15^ To precisely evaluate the transcripts produced from each allele in iPSC-derived cardiomyocytes (iPSC-CMs), polymerase chain reaction (PCR) probes specifically targeting wild type (WT) or Δ160E transcripts were generated. Droplet digital PCR (ddPCR) analysis using cDNA obtained from patient-derived iPSC-CMs revealed comparable amounts of transcripts from the WT and Δ160E alleles (Figure 2A), indicating that Δ160E acts as a gain-of-function mutation. We then sought to generate allele-modified iPSC clones to precisely evaluate the pathological phenotype of the *TNNT2* Δ160E mutation under the same genetic background using CRISPR/Cas9 genome editing. Using homology-directed repair, we corrected the mutation in patient-derived iPSCs with heterozygous Δ160E and introduced a homozygous mutation to enhance the pathological phenotype of Δ160E. We generated a pX459 vector encoding gRNA_*TNNT2*, targeted just upstream of the repeated GAG sequences in exon 12 of *TNNT2* (Figure 2B). The Cel-I assay showed that gRNA_*TNNT2* efficiently cleaved the endogenous targeted genomic sequence of *TNNT2* (Figure 2C). Next, we generated homology-directed repair template DNA containing WT and Δ160E *TNNT2* sequences flanked with 959 bp 5´- and 642 bp 3´-homology arms and introduced synonymous mutations around the protospacer adjacent motif sequence to avoid Cas9-mediated re-cleavage (Figure 2B). The pX459 vector encoding gRNA_*TNNT2* combined with the generated homology-directed repair template vector was transfected into the patient-derived iPSCs, and the allele-modified clone was screened by Sanger sequencing. Finally, after repeated sessions of genome editing, we obtained an iPSC clone with a homozygously corrected allele (WT-iPSCs) and those homozygous for the Δ160E allele (Homo-Δ160E-iPSCs) using homology-directed repair (Figure 2D; Figure S1D). We also obtained an iPSC clone carrying the unchanged heterozygous Δ160E *TNNT2* sequence during the same sibselection procedure as a control (Hetero-Δ160E-iPSCs). These isogenic iPSC clones uniformly expressed pluripotent markers, exhibited normal karyotypes, and had the ability to differentiate into the 3 germ layers (Figure S1A, S1B, and S1C). The generated Homo-Δ160E-, Hetero-Δ160E-, and WT-iPSCs were differentiated into cardiomyocytes (Figure 2E). Spontaneous beating was similarly observed on day 10 in these iPSC-CMs after inducing differentiation, and the efficiency of differentiation evaluated by flow cytometry using anti-TnT antibody was comparable among the set of isogenic iPSC-CMs (Figure 2F). These iPSC-CMs showed no apparent difference in sarcomere structure by immunostaining and protein expression levels of TnT and α-actinin (Figure 2G through 2I). The differentiated iPSC-CMs were replated once into plastic 6-well plates. We then initiated electrical stimulation at 2 Hz and continued it for 1 week to promote maturation.^16^ On day 21, iPSC-CMs were replated into 24-well plates or 96-well imaging plates (Greiner) and used for further analysis (Figure 2E).

### *TNNT2* Δ160E Mutation Promoted Sarcomeric Calcium Retention in iPSC-CMs

**Figure 3.**
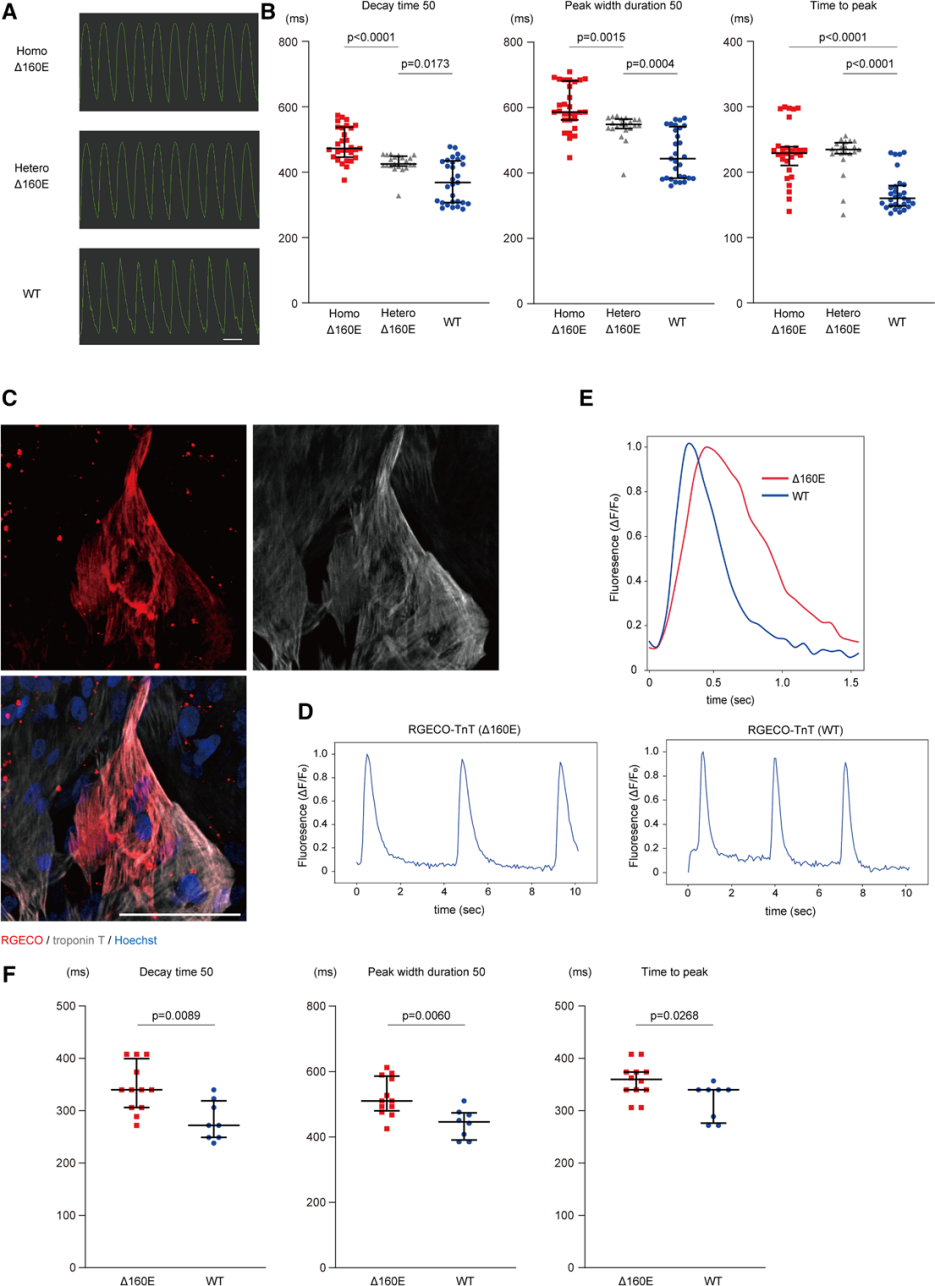
**TNNT2 Δ160E mutation promoted sarcomeric calcium retention in induced pluripotent stem cell–derived cardiomyocytes (iPSC-CMs). A**, Representative recordings of the Cal-520 am fluorescence signal at a pacing rate of 1 Hz in the set of isogenic iPSC-CMs. Homo- and Hetero-Δ160E-iPSC-CMs exhibited an obtuse waveform compared with the wild type (WT)-iPSC-CMs. Scale bar: 1 s. **B**, Extracted parameters of calcium transients were analyzed in the set of isogenic iPSC-CMs (number of analyzed wells: Homo-Δ160E-iPSC-CMs, 29; Hetero-Δ160E-iPSC-CMs, 21; WT-iPSC-CMs, 29; from n=5 technical replicates). The data are shown as Dot plots. **C**, iPSC-CMs replated in 96-well plates at 2 weeks after differentiation were transduced with AAV2-RGECO-*TNNT2*. Seven days after transduction, iPSC-CMs were fixed and immunostained with the indicated antibodies. The RGECO signal (red color) co-localized with the TnT signal (white color) in iPSC-CMs is shown. Scale bar: 100 μm. **D**, Raw waveform of myofilament-localized calcium transients in iPSC-CMs expressing the RGECO-TnT fusion protein. **E**, Representative waveforms of myofilament-localized calcium transients in iPSC-CMs expressing RGECO-TnT (Δ160E: red and WT: blue). **F**, The extracted parameters were analyzed in iPSC-CMs expressing RGECO-TnT (Δ160E, n=12 cells) and RGECO-TnT (WT, n=8 cells). Each data point is shown as a Dot plot. PCR indicates colymerase chain reaction.

We measured the calcium transients in these isogenic iPSC-CMs at 4 to 5 weeks after differentiation by FDSS/μCELL using Cal-520 am as the calcium indicator (Figure 3A). Under electrical stimulation at 1 Hz, the *TNNT2* Δ160E mutation dose-dependently prolonged the decay time 50 (472 [446–537] ms in Homo-Δ160E-iPSC-CMs, 425 [419–449] ms in Hetero-Δ160E-iPSC-CMs, and 368 [292–435] ms in WT-iPSC-CMs), and peak width duration 50 (586 [562–682] ms in Homo-Δ160E-iPSC-CMs, 549 [536–565] ms in Hetero-Δ160E-iPSC-CMs, and 444 [384–541] ms in WT-iPSC-CMs; Figure 3B). The time to peak was significantly prolonged in Homo- (229 [210–239] ms) and Hetero-Δ160E-iPSC-CMs (235 [228–245] ms) than in the WT-iPSC-CMs (160 [148–180] ms). These data suggest that the *TNNT2* Δ160E mutation impaired cytosolic calcium decline in iPSC-CMs, consistent with previous findings demonstrating that the rates of calcium decline are decreased and that the time to 90% calcium decline is increased in cardiomyocytes isolated from murine hearts overexpressing TnT Δ160E.^12^ Next, we used the red fluorescent genetically encoded calcium indicators for optical imaging-TnT fusion protein^17^ to directly visualize calcium changes in the sarcomere. Using this method, we could precisely extract localized sarcomeric calcium handling in iPSC-CMs. We generated the AAV2 (adeno-associated virus-2) encoding red fluorescent genetically encoded calcium indicators for optical imaging (RGECO)-*TNNT2* driven by the cytomegalovirus promoter (AAV2-RGECO-*TNNT2*; Figure S2A) because AAV2 can transduce human iPSC-CMs with high efficiency.^18,19^ iPSC-CMs without *TNNT2* mutation were transduced with AAV2-RGECO-*TNNT2* (WT or Δ160E) and red fluorescent signals from the RGECO-TnT fusion protein were analyzed using a confocal microscope (Spin SR). Immunostaining confirmed that the RGECO-TnT fusion protein co-localized with the endogenous TnT in sarcomeres (Figure 3C). Overexpression analysis using the RGECO-TnT fusion protein clarified that the Δ160E mutation prolonged the decay time 50 (340 [306–400] ms in Δ160E and 272 [249–319] ms in WT), peak width duration 50 (510 [480–587] ms in Δ160E and 446 [391–474] ms in WT), and time to peak (360 [340–374] ms in Δ160E and 340 [276–340] ms in WT) of the myofilament-localized calcium transients in iPSC-CMs (Figure 3D through 3F). These data, combined with the calcium transient analysis in isogenic iPSC-CMs, indicated that the *TNNT2* Δ160E mutation promoted sarcomeric calcium retention in iPSC-CMs.

The prolonged decay time 50 and peak width duration were similarly observed in iPSC-CMs transduced with AAV2 encoding RGECO-*TNNT2* with R92W mutation, but not in iPSC-CMs transduced with AAV2 encoding RGECO-*TNNT2* with E163K mutation or *MYL2*-RGECO with R58Q mutation (Figure S2B). These results suggest that sarcomeric calcium retention is a specific pathological phenotype caused by the genetic mutations defined as pathogenic variants (R92W and Δ160E in *TNNT2*),^20^ consistent with the previous report using RGECO-TnT with R92Q mutation.^17^

### iPSC-CMs With Homozygous and Heterozygous Δ160E Mutation Exhibited Relaxation Impairment in Contractile Dynamics and Arrhythmic Phenotypes

**Figure 4.**
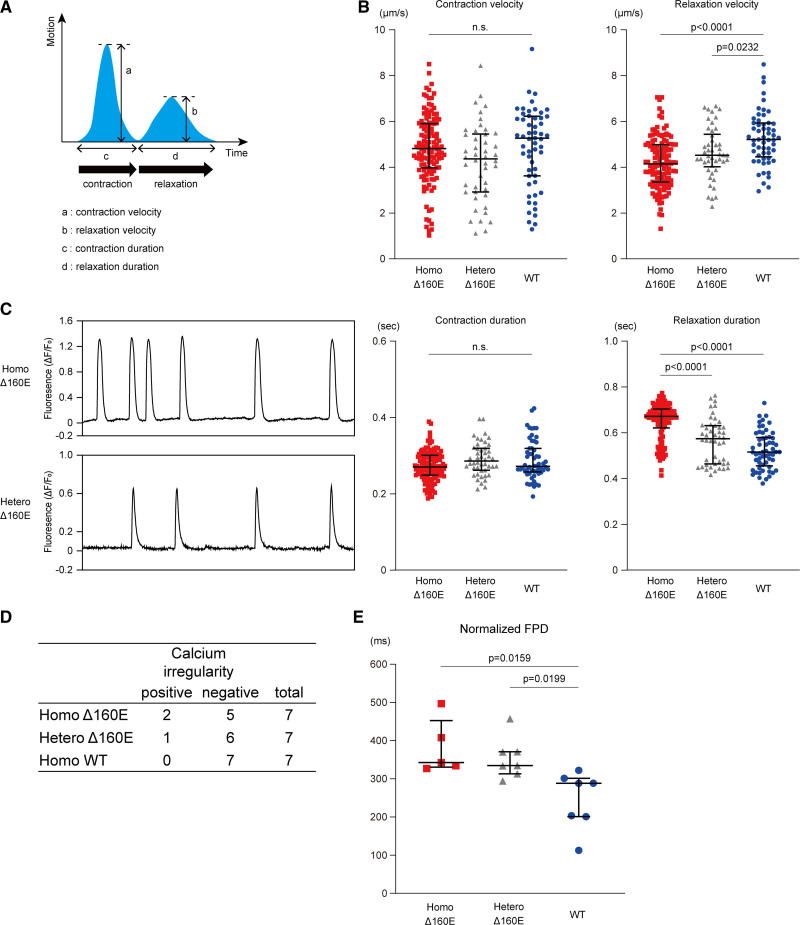
**Induced pluripotent stem cell–derived cardiomyocytes (iPSC-CMs) with homozygous and heterozygous Δ160E mutation exhibited relaxation impairment in contractile dynamics and arrhythmic phenotypes. A**, Schematic diagram of contraction and relaxation motion waveforms obtained using a cell motion imaging system. **B**, Extracted parameters of contractile properties were analyzed in the set of isogenic iPSC-CMs (number of analyzed ROIs: Homo-Δ160E-iPSC-CMs, 126; Hetero-Δ160E-iPSC-CMs, 46; wild type (WT)-iPSC-CMs, 57; from n=5 technical replicates). Data are shown as Dot plots. **C**, Representative waveform of irregular calcium transients in the isogenic iPSC-CMs. Each recording duration was 1 minute. **D**, The incidence of calcium irregularity in the isogenic iPSC-CMs from 7 sessions in each clone. **E**, The normalized FPD was analyzed in the isogenic iPSC-CMs (number of analyzed samples: Homo-Δ160E-iPSC-CMs, 5; Hetero-Δ160E-iPSC-CMs, 7; WT-iPSC-CMs, 7). Data are shown as Dot plots.

To evaluate the effect of retained calcium concentration on contraction or relaxation in iPSC-CMs, we recorded a phase-contrast image of isogenic iPSC-CMs at 4 to 5 weeks after differentiation using a high-speed, camera-based, motion analysis system (Figure 4A). Homo- and Hetero-Δ160E-iPSC-CMs exhibited significantly decreased relaxation velocity (4.15 [3.36–4.99] μm/s, *P*<0.0001 and 4.53 [4.02–5.44] μm/s, *P*=0.0232, respectively) compared with that in WT-iPSC-CMs (5.21 [4.46–5.93] μm/s), whereas the contraction velocity showed no significant difference among these isogenic iPSC-CMs (Figure 4B). Furthermore, the relaxation duration was especially prolonged in Homo-Δ160E-iPSC-CMs (672 [621–704] ms) than in the other isogenic iPSC-CMs (574 [464–632] ms in Hetero-Δ160E-iPSC-CMs, *P*<0.0001 and 516 [456–580] in WT-iPSC-CMs, *P*<0.0001). There was no significant difference in contraction duration among the isogenic iPSC-CMs. Contraction and relaxation deformation distance defined by motion vector analysis represent the contractile and relaxation forces,^21^ and these isogenic iPSC-CMs showed comparable values in the contraction and relaxation deformation distances (Figure S3A). Our experimental data regarding impaired relaxation were consistent with previous findings demonstrating that the peak rate relaxation is decreased and time to 90% relaxation is increased in cardiomyocytes isolated from murine hearts overexpressing TnT Δ160E.^12^ Although isolated cardiomyocytes from Δ160E transgenic mice showed dose-independent relaxation impairment, our data using human isogenic iPSC-CMs exhibited dose-dependent relaxation impairment caused by the *TNNT2* Δ160E mutation.

To evaluate the arrhythmic phenotype of Δ160E mutation as observed in the proband, we investigated calcium irregularities in the isogenic iPSC-CMs using FDSS/μCELL. Under electric field stimulation at 1 Hz, these iPSC-CMs did not show irregular calcium transients. During the 1-minute recording on spontaneous calcium transients, irregular calcium transient was detected only in Homo- and Hetero-Δ160E-iPSC-CMs, but not in WT-iPSC-CMs (n=2/7 analyzed sessions in Homo-Δ160E-iPSC-CMs, n=1/7 in Hetero-Δ160E-iPSC-CMs, n=0/7 in WT-iPSC-CMs; Figure 4C and 4D). Electrical activity measurements using multielectrode array (MEA) data acquisition system^22,23^ demonstrated that only Homo-Δ160E-iPSC-CMs exhibited abnormal electrical activity during the recording period (Figure S3B). Of note, Homo- and Hetero-Δ160E-iPSC-CMs showed significantly prolonged field potential duration normalized to the beating rate (343 [331–453] ms, *P*=0.0159 and 335 [313–371] ms, *P*=0.0199, respectively) compared with that in WT-iPSC-CMs (288 [201–301] ms; Figure 4E). These data suggest that isogenic iPSC-CMs with Δ160E mutation recapitulated the arrhythmic phenotypes observed in the proband.

### *TNNT2* Δ160E Mutation Accelerated the Nuclear Translocation of NFAT and Caused Hypertrophy in iPSC-CMs

**Figure 5.**
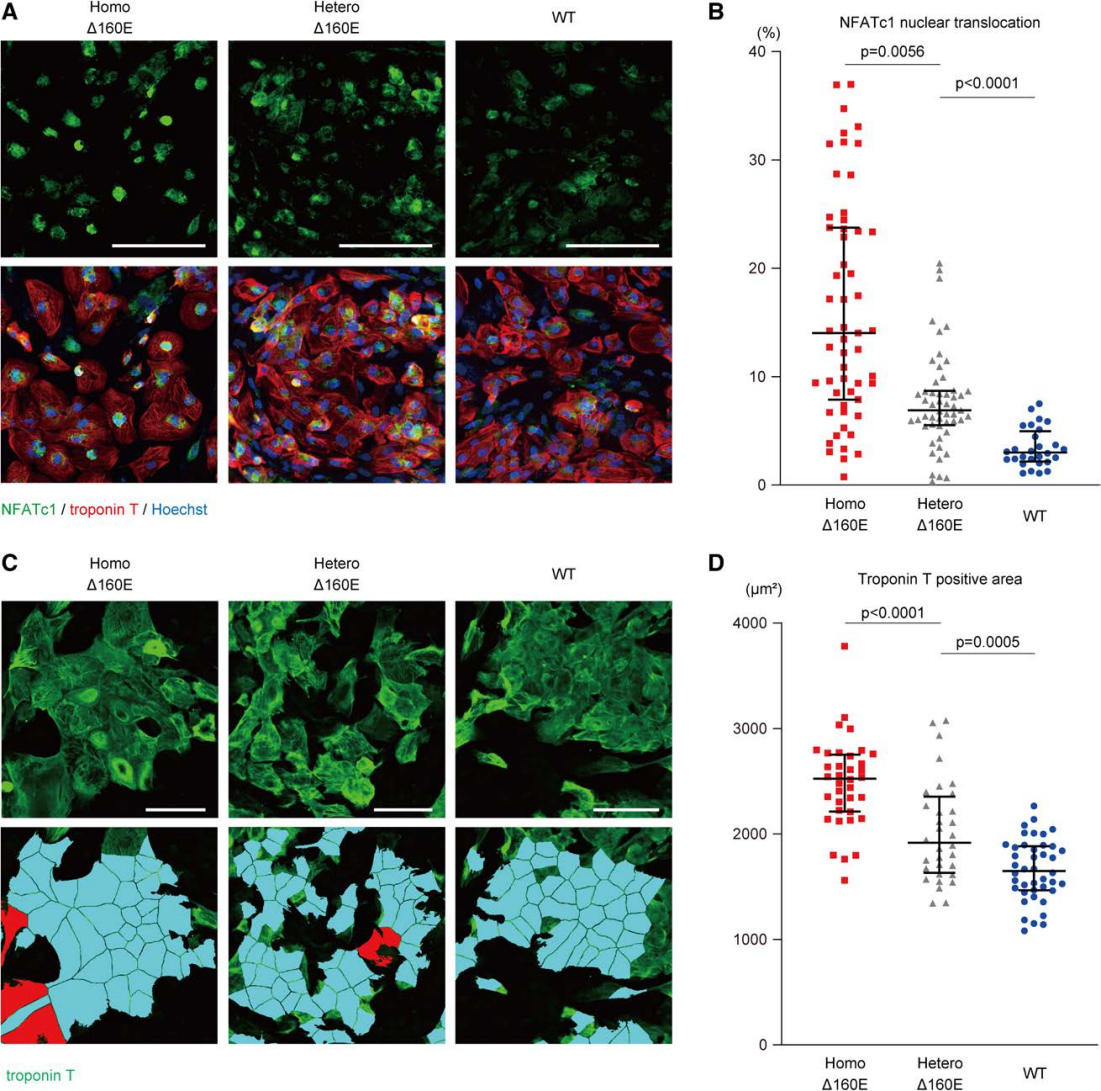
**TNNT2 Δ160E mutation accelerated the nuclear translocation of NFATc1 and caused hypertrophy in induced pluripotent stem cell–derived cardiomyocytes (iPSC-CMs). A**, iPSC-CMs replated in 96-well plates were fixed at 4 to 5 weeks after differentiation and immunostained with the indicated antibodies. Representative fluorescence images of the NFATc1-positive signal (upper) and merged images (below) in the set of isogenic iPSC-CMs are shown. Nuclei were detected by Hoechst staining. Scale bar: 50 μm. **B**, The images shown in **A** were quantitatively analyzed using a high-content imaging system (number of analyzed cells: Homo-Δ160E-iPSC-CMs, 33252; Hetero-Δ160E-iPSC-CMs, 29427; wild type (WT)-iPSC-CMs, 21572; from n=5 technical replicates, 25 images in each experiment). The data are shown as Dot plots. **C**, The set of isogenic iPSC-CMs replated in 96-well plates was fixed at 4 to 5 weeks after differentiation and immunostained with the indicated antibody. Nuclei were detected using Hoechst staining. The TnT-positive area was indicated with a light blue color, and a red colored lesion was excluded from the analysis. Scale bar: 50 μm. **D**, The images shown in **C** were quantitatively analyzed using a high-content imaging system (number of analyzed cells: Homo-Δ160E-iPSC-CMs, 13960; Hetero-Δ160E-iPSC-CMs, 15208; WT-iPSC-CMs, 27258; from n=4 technical replicates, 25 images in each experiment). Data are shown as Dot plots.

Sustained elevation of intracellular calcium could accelerate the nuclear translocation of NFATc1 (nuclear factor of activated T-cells c1).^24^ Therefore, we investigated the nuclear translocation of NFATc1 in isogenic iPSC-CMs at 4 to 5 weeks after differentiation by analyzing their immunofluorescence signals with a high-content imaging system, as described previously.^25,26^ NFATc1 nuclear translocation was defined as the NFATc1 fluorescent signal merged with the Hoechst signal in iPSC-CMs. The *TNNT2* Δ160E mutation dose-dependently accelerated the nuclear translocation of NFATc1 (14.0 [7.88–23.8]% in Homo-Δ160E-iPSC-CMs, 6.90 [5.53–8.69]% in Hetero-Δ160E-iPSC-CMs, and 3.01 [2.18–4.96]% in WT-iPSC-CMs), indicating that the *TNNT2* Δ160E mutation promoted the hypertrophic signaling pathway (Figure 5A and 5B). We then assessed the hypertrophic phenotype of iPSC-CMs with homozygous and heterozygous Δ160E mutations. Cell size was measured in Homo-Δ160E- and Hetero-Δ160E- and WT-iPSC-CMs at 4 to 5 weeks after differentiation. The *TNNT2* Δ160E mutation promoted the enlargement of cell size, defined as a TnT-positive area, in a dose-dependent manner (2526 [2213–2753] μm^2^ in Homo-Δ160E-iPSC-CMs, 1917 [1632–2354] μm^2^ in Hetero-Δ160E-iPSC-CMs, and 1649 [1467–1885] μm^2^ in WT-iPSC-CMs; Figure 5C and 5D).

### Calcium Dysregulation in Δ160E-iPSC-CMs Promoted Phosphorylation of Thr17 of Phospholamban and CaMKIIδ

**Figure 6.**
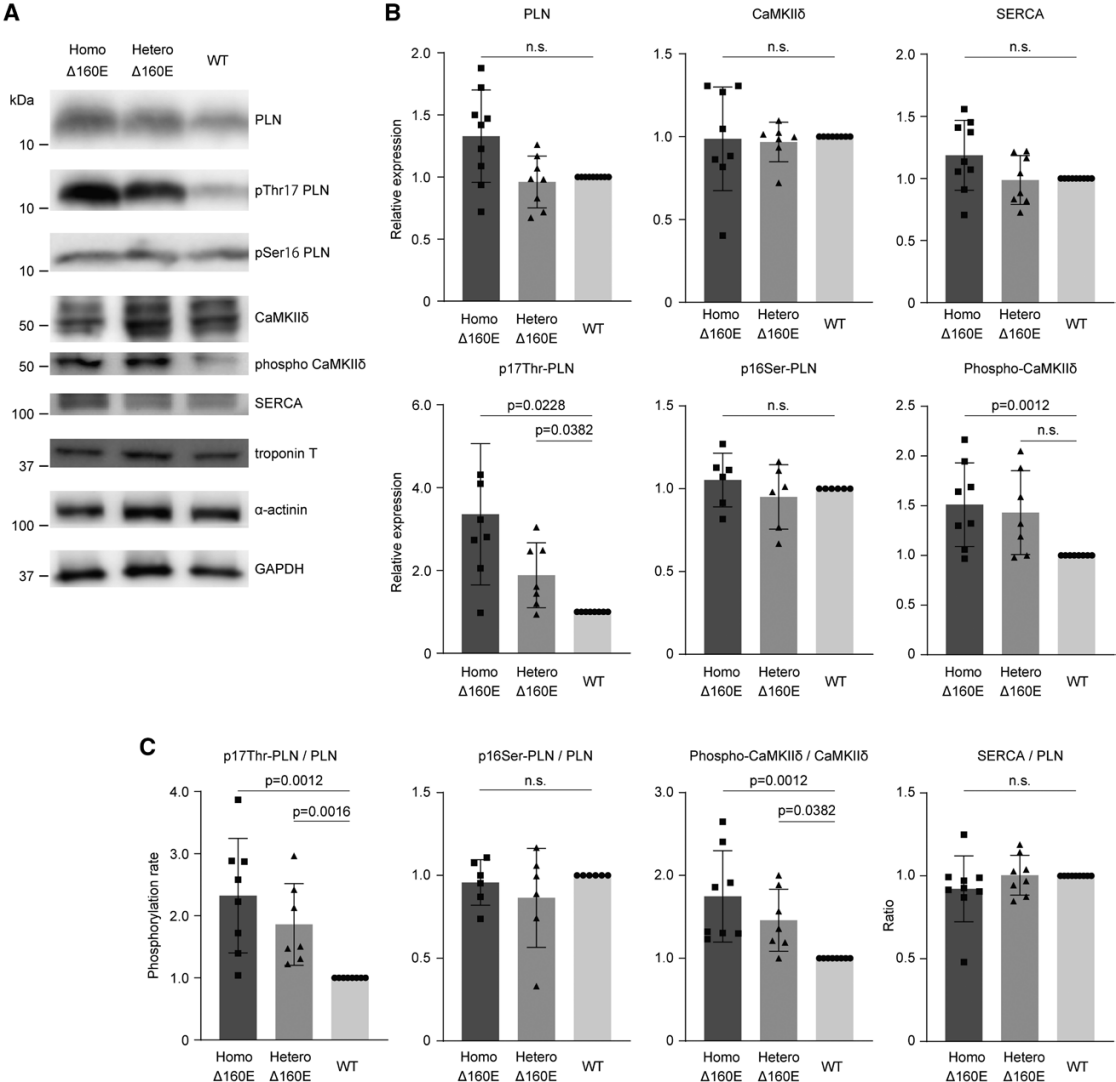
**Calcium dysregulation in Δ160E-induced pluripotent stem cell–derived cardiomyocytes (iPSC-CMs) promoted phosphorylation of Thr17 of phospholamban and CaMKIIδ. A**, Whole-cell lysates were extracted from the set of isogenic iPSC-CMs at 4 to 5 weeks and were analyzed by Western blotting using the indicated antibodies. Representative western blots of the set of isogenic iPSC-CMs are shown. **B**, Graphs summarizing the western blot results. Quantified protein expression levels normalized by GAPDH expression were compared among the 3 groups (n=9, mean±SD). **C**, The phosphorylation rate of PLN (phospholamban) at Thr17 and Ser16 was calculated by dividing the expression level of phosphorylated PLN at Thr17 and Ser16 by the total PLN. The ratio of phosphorylated CaMKIIδ (calcium/calmodulin-dependent protein kinase IIδ) to total CaMKIIδ was calculated to determine the phosphorylation rate. The ratio of SERCA (sarcoplasmic reticulum calcium ATPase) to PLN was calculated by dividing the SERCA expression level by the total PLN (n=9, mean±SD). GAPDH indicates glyceraldehyde-3-phosphate dehydrogenase.

Retention of calcium caused by *TNNT2* Δ160E mutation in iPSC-CMs potentially affects downstream mechanisms including the expression levels of calcium regulatory proteins or phosphorylation state of PLN (phospholamban), which regulates calcium reuptake into the sarcoplasmic reticulum.^27^ We investigated the expression levels or phosphorylation state of calcium regulatory proteins in the isogenic set of iPSC-CMs at 4 to 5 weeks after differentiation (Figure 6A). Repeated experiments showed that the expression levels of PLN, SERCA (sarcoplasmic reticulum calcium ATPase), and the resulting SERCA/PLN ratio were not significantly different among isogenic iPSC-CMs (Figure 6A through 6C). Notably, both Homo- and Hetero-Δ160E-iPSC-CMs exhibited a significantly increased phosphorylation rate of PLN, specifically at Thr17, the CaMKIIδ (calcium/calmodulin-dependent protein kinase IIδ) phosphorylation site, compared with WT-iPSC-CMs (Figure 6A through 6C). However, there was no significant difference in the phosphorylation rate of PLN at Ser16, a PKA (protein kinase A) phosphorylation site, among these isogenic iPSC-CMs. The phosphorylation rate of CaMKIIδ was significantly increased in Homo- and Hetero-Δ160E-iPSC-CMs (Figure 6A through 6C), suggesting a compensatory change against the retained cytosolic calcium due to the *TNNT2* Δ160E mutation. Specifically, repeated experiments using isogenic iPSC-CMs revealed a dose-dependent increase in the phosphorylation rate of PLN at Thr17 and CaMKIIδ due to the *TNNT2* Δ160E mutation (Figure 6B and 6C). In *Tnnt2* Δ160E transgenic mice, overexpression of TnT with 70% expression compared with endogenous TnT increased both the Ser16 and Thr17 phosphorylation of PLN,^12^ suggesting that our disease model using isogenic human iPSC-CMs may facilitate detection of downstream effects caused by the *TNNT2* Δ160E mutation-mediated calcium dysregulation.

### Epigallocatechin-3-Gallate Recovered the Augmented Calcium Retention Caused by the *TNNT2* Δ160E Mutation in iPSC-CMs

**Figure 7.**
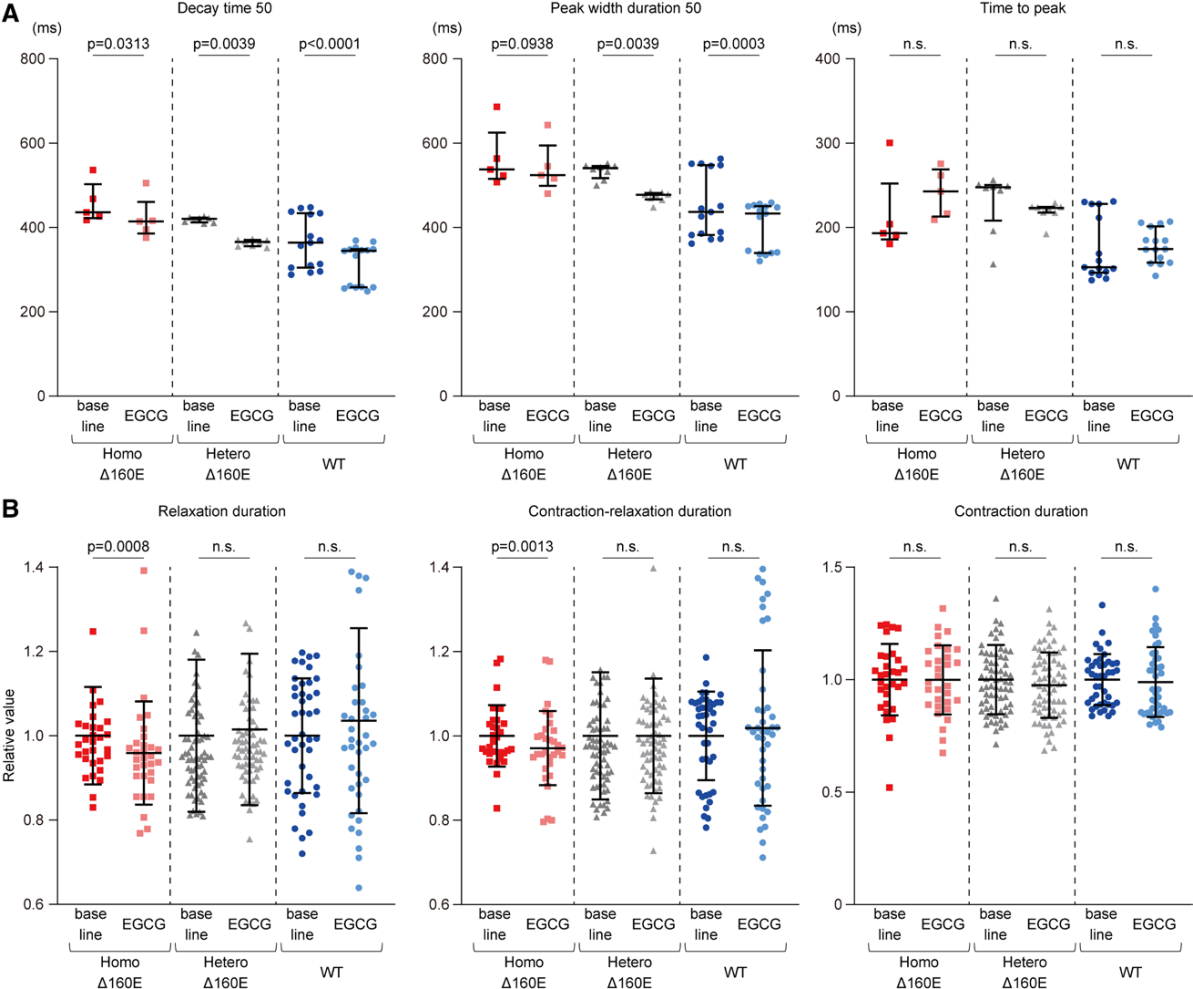
**EGCG shortened decay time in calcium transient and relaxation duration in induced pluripotent stem cell–derived cardiomyocytes (iPSC-CMs) with Δ160E mutation. A**, The effect of epigallocatechin-3-gallate (EGCG) on calcium transients was assessed in the set of isogenic iPSC-CMs (number of analyzed wells: Homo-Δ160E-iPSC-CMs, 5; Hetero-Δ160E-iPSC-CMs, 8; wild type (WT)-iPSC-CMs, 15; from n=5 technical replicates). Extracted parameters just before and 10 minutes after the addition of EGCG were analyzed in each group. Data are shown as Dot plots. **B**, The effect-of EGCG on each parameter of contractile properties was assessed in the set of isogenic iPSC-CMs (number of analyzed ROIs: Homo-Δ160E-iPSC-CMs, 31; Hetero-Δ160E-iPSC-CMs, 68; WT-iPSC-CMs, 42; from n=6 technical replicates). Each parameter was plotted as relative value compared with baseline in each group. Data are shown as Dot plots.

Epigallocatechin-3-gallate (EGCG), a major catechin in green tea, had been reported to interact with a binding site for cardiac troponin C and to decrease calcium sensitivity.^28,29^ In vivo experimental models and clinical trials have demonstrated that EGCG improves diastolic dysfunction by correcting the calcium hypersensitivity of cardiac myofilaments in animal models and in human patients with cardiomyopathy.^28,30^ Thus, we assessed the effect of EGCG on calcium transients by FDSS/μCELL using the set of isogenic iPSC-CMs at 4 to 5 weeks after differentiation. EGCG at 100 μmol/L significantly decreased the decay time 50 in Homo-Δ160E- (414 [385–461] versus 435 [421–501] ms; *P*=0.0313), Hetero-Δ160E- (366 [356–371] versus 419 [410–422] ms; *P*=0.0039), and WT-iPSC-CMs (344 [258–348] versus 363 [303–433] ms; *P*<0.0001) compared with baseline values (Figure 7A). In contrast, EGCG did not significantly affect the time to peak in the calcium transients of any isogenic iPSC-CM. The peak width duration 50 was shortened in the Hetero-Δ160E-iPSC-CMs (477 [467–482] versus 539 [516–545] ms; *P*=0.0039) and WT-iPSC-CMs (433 [340–450] versus 436 [381–547] ms; *P*=0.0003), with a trend observed in Homo-Δ160E-iPSC-CMs (524 [498–594] versus 536 [514–624] ms; *P*=0.0938). Importantly, after adding EGCG, Homo- and Hetero-Δ160E-iPSC-CMs showed comparable values of decay time 50 and peak width duration 50 to those of WT-iPSC-CMs at baseline. These data provided the proof of concept that EGCG, a calcium desensitizer, rescued the upstream pathological phenotype caused by the *TNNT2* Δ160E mutation in human cardiomyocytes.

Regarding the effect on contractile properties, EGCG significantly shortened the relaxation duration and contraction-relaxation duration in Homo-Δ160E-iPSC-CMs (558 [533–579] versus 581 [554–606] ms; *P*=0.0008, and 810 [786–852] versus 830 [807–873] ms; *P*=0.0013) compared with baseline value, which had been significantly prolonged than that in the other isogenic iPSC-CMs (Figure 7B). In Hetero-Δ160E- and WT-iPSC-CMs, EGCG did not significantly alter the relaxation duration and contraction-relaxation duration. Regarding contraction duration, there were no significant difference between baseline values and those after adding EGCG in each clone.

## Discussion

We encountered a Japanese case of familial HCM with *TNNT2* Δ160E mutation, who developed advanced heart failure with left ventricular systolic dysfunction > 20 years after the initial diagnosis of HCM. Although the proband received several pharmacological (beta blocker, angiotensin II receptor blocker, and anti-arrhythmic drugs) and nonpharmacological (cardiac resynchronization) therapies, these standard interventions did not improve or prevent disease progression. Furthermore, because the deleterious effect of *the TNNT2* Δ160E mutation is not lethal, it gradually affects the patient’s heart over several decades; thus, the rare *TNNT2* Δ160E mutation may be inherited without early clinical diagnosis, resulting in worldwide prevalence.^6–9^ These clinical characteristics require a convenient human disease model to recapitulate the pathological phenotype caused by the *TNNT2* Δ160E mutation for therapeutic development.

Droplet digital PCR analysis using iPSC-CMs generated from the proband indicated that the Δ160E mutation does not affect the stability of mutated transcripts and acts as a gain-of-function mutation, suggesting that the homozygous Δ160E mutation further facilitates the pathological phenotype. In fact, *TNNT2* Δ160E mutation in iPSC-CMs exhibited prolonged calcium decay, relaxation impairment, hypertrophy, nuclear translocation of NFATc1, and dysregulation of calcium regulatory proteins in a dose-dependent manner. The Δ160E mutation is located in the linker region of TnT and decreases flexibility of TNT1.^10^ The atomistic models and biochemical analyses have revealed that Δ160E disturbed calcium dissociation from troponin C and increased calcium sensitivity.^11^ Importantly, overexpression analysis using the RGECO-TnT fusion protein revealed that the Δ160E mutation augmented sarcomeric calcium retention in iPSC-CMs. Interestingly, Δ160E rather than E163K mutation affected myofilament-localized calcium transients, although both mutations are located at the consecutive 4 E amino acids at linker region of TnT. These data suggest that deletion rather than replacement of a single amino acid at the adjacent location of the linker has more severe impact on TnT function. These data, combined with previous findings, suggest that our established Hetero- and Homo-Δ160E-iPSC-CMs can serve as useful disease models to recapitulate the increased calcium affinity of troponin C and augmented calcium retention as a major upstream molecular basis of pathology in human cardiomyocytes. Furthermore, Homo-Δ160E-iPSC-CMs recapitulated the distinct nuclear translocation of NFATc1. Retained cytosolic calcium due to the Δ160E mutation could activate calcineurin, a serine/threonine-specific phosphatase that plays a crucial role in the development of cardiac hypertrophy.^31^ Calcineurin dephosphorylates NFATc1 and promotes translocation from the cytosol into the nucleus, which accelerates pro-hypertrophic gene expression.^32^ Therefore, nuclear signals raised from NFATc1 in Homo-Δ160E-iPSC-CMs represents the direct downstream change caused by retained calcium and can be the useful readout for therapeutic screening.

Dose-dependent increase in phosphorylation of CaMKIIδ and PLN at Thr17 was observed in Homo- and Hetero-Δ160E-iPSC-CMs. In human and animal model, CaMKIIδ activation is reported to be responsible for the pathogenesis of disrupted calcium regulation and disease progression in HCM.^12,33^ CaMKIIδ is activated by increased intracellular calcium levels and phosphorylates multiple proteins associated with excitation-contraction coupling, including PLN.^34^ PLN inhibits SERCA and decreases calcium reuptake into the sarcoplasmic reticulum. CaMKIIδ and PKA decrease the inhibitory activity of PLN and accelerate calcium reuptake by phosphorylation at Thr17 and Ser16, respectively.^27^ In a transgenic mouse model, phosphorylation of both Ser16 and Thr17 of PLN was increased in mice with 70% expression of Δ160E TnT compared with that in mice with lower expression.^12^ In our isogenic iPSC-CMs, the *TNNT2* Δ160E mutation promoted phosphorylation of PLN specifically at the Thr17 residue and CaMKIIδ activation in a dose-dependent manner. This suggests that our disease model may capture downstream compensatory changes against calcium retention.

EGCG, a small compound that binds the C-lobe of troponin C and causes calcium desensitization of cardiac myofilaments,^28,29^ shortened the prolonged calcium decay and relaxation duration in Δ160E-iPSC-CMs. Our results were consistent with the previous literatures demonstrating that EGCG decreased calcium sensitivity in skinned cardiac muscle fibers isolated from Δ160E *Tnnt2* transgenic mice^28^ and was recognized as a promising compound restoring the impaired cardiac function through its direct calcium-desensitizing effects.^35^ EGCG can be a promising drug to prevent disease pathogenesis in patients with HCM carrying the *TNNT2* Δ160E mutation.

In our human disease model, the Δ160E mutation promoted the activation of both calcineurin and CaMKIIδ. Calcineurin-NFAT signaling is known to be responsible for pathological cardiac hypertrophy.^36^ Although CaMKIIδ activation is thought to be a compensatory change and has an inhibitory effect on calcineurin, chronic CaMKIIδ activation may cause adverse remodeling and disease progression.^34^ Thus, a therapeutic approach to suppress the excessive activity of both calcineurin and CaMKIIδ is needed to prevent disease progression in the Δ160E mutation.

To the best of our knowledge, this is the first report of a human model using patient-derived iPSC-CMs that recapitulated pathological phenotypes caused by Δ160E mutation in *TNNT2* under an isogenic background and that indicated the therapeutic possibility of EGCG in patients with HCM carrying Δ160E. Previous reports investigated pathological phenotype using transgenic mice expressing the Δ160E mutant TnT.^12^ However, cautions are needed when applying these results to the human diseases because there could be interspecies differences.^37^

This study has some limitations. We could not recapitulate the systolic dysfunction developed in the proband with the heterozygous Δ160E mutation because of the immaturity of iPSC-CMs. Several methods including formation of 3-dimensional heart tissue and chronic electrical stimulation are thus needed to accelerate maturity.^24,38^

In conclusion, isogenic iPSC-CMs recapitulate prolonged calcium decay, relaxation impairment, and subsequent calcium-regulated signaling pathways caused by the *TNNT2* Δ160E mutation. EGCG reversed calcium retention and could thus be a promising drug to prevent disease pathogenesis caused by the *TNNT2* Δ160E mutation. This study using a combination of disease model iPSC-CMs and genome editing technology could thus provide a useful human cellular model for understanding pathogenic molecular mechanisms and for developing therapeutic approaches to prevent HCM pathology.

## Article Information

### Acknowledgments

We thank M. Moriyasu for technical assistance. This study was supported by Center of Medical Innovation and Translational Research, and Center for Medical Research and Education in Graduate School of Medicine, Osaka University.

### Sources of Funding

This work was supported by JSPS KAKENHI Grant Numbers 19K08489, 19K16518, 20K21602, 21H02915, grants-in-aid from the Japanese Ministry of Health, Labor, and Welfare, the Japan agency for medical research and development (19bm0804008h0003). This work was also supported by the Cell Science Research Foundation and the Grant for Basic Research of the Japanese Circulation Society (2018), SENSHIN Medical Research Foundation, Novartis Pharma, Takeda Pharmaceutical Company, Lilly, MSD, Daiichi Sankyo, Mitsubishi Tanabe Pharma.

### Disclosures

Department of Medical Therapeutics for Heart Failure is a Joint Research Department with TOA EIYO Pharmaceutical Company.

### Supplemental Material

Supplemental Methods

Table S1

Figures S1–S3

References^39–49^

## Supplementary Material


