# Supplementary material for: Human-Induced Pluripotent Stem Cell–Derived Cardiomyocyte Model for TNNT2 Δ160E-Induced Cardiomyopathy
Source: Circ Genom Precis Med. 2022 Jul 12;15(5):e003522. doi: 10.1161/CIRCGEN.121.003522 (PMC9584061; doi:10.1161/CIRCGEN.121.003522)
Supplement: Supplementary file 1 [file hcg-15-e003522-s001.pdf]

SUPPLEMENTAL MATERIAL

Supplemental Methods

Human samples

We collected clinical data and samples from patients who were admitted to Osaka University Hospital. The use of human samples and the genomic analysis were approved by the Ethics Committee of Osaka University Hospital, and written informed consent was obtained from the participant. This investigation conformed to Ethical Guidelines for Medical and Health Research Involving Human Subjects in Japan and all principles outlined in the Declaration of Helsinki.

Reagents and antibodies

Reagents and antibodies used in this study:

Oct-3/4 (C-10) (Santa Cruz Biotechnology, Dallas, Texas, USA, Cat# sc-5279, RRID: AB_628051), TRA-1-60 (Merck Millipore, Burlington, Massachusetts, USA, Cat# MAB4360, RRID: AB_2119183), SSEA-4 (Merck Millipore, Cat# MAB4304, RRID: AB_177629), Nanog (Abcam, Cambridge, MA, USA, Cat# ab80892, RRID: AB_2150114), Troponin T-C (CT3) (Santa Cruz Biotechnology, Cat# sc-20025, RRID: AB_628403) (immunostaining and western blot), Troponin T (Abcam Cat# ab45932, RRID:AB_956386) (immunostaining), GAPDH (Santa Cruz Biotechnology Cat# sc-47724, RRID: AB_627678), Sarcomeric Alpha Actinin (EA-53) (Abcam, Cat# ab9465, RRID: AB_307264), Phospholamban (Cell Signaling Technology Cat# 14562, RRID: AB_2798511), Phospho-Thr17-phospholamban (Badrilla Cat# A010-13AP, RRID: AB_2617048), Phospho-Ser16-phospholamban (Badrilla Cat# A010-12AP, RRID: AB_2617047), CaMKIIδ (Santa Cruz Biotechnology Cat# sc-100362, RRID:

AB_2068097), Phospho-CaMKIIβδγ (Thr287) (Thermo Fisher Scientific Cat# PA5-37833, RRID: AB_2554441), SERCA (Abcam Cat# ab3625, RRID:AB_303961), NFAT2 (Abcam Cat# ab25916, RRID:AB_448901), Hoechst 33342 (DOJINDO Molecular Technologies, Kumamoto, Japan, Cat# H342), BV421 Mouse IgG1, k Isotype Control (BD Bioscience, Tokyo, Japan, Cat# 562438, RRID: AB_2721018), BV421 Mouse Anti-Cardiac Troponin T (BD Bioscience, Cat# 565618, RRID: AB_2739306) (flow cytometry), Puromycin dihydrochloride (SIGMA, Cat# P9620-10ML), (-)-Epigallocatechin-3-gallate (Fujifilm Cat# 059-05411).

Amplicon sequence analysis

Genetic screening was performed as described previously.[14] The genomic DNA was extracted from peripheral blood of the proband and that of his three daughters using QIAamp DNA mini kit (QIAGEN) and analyzed by Ion Ampliseq Cardiovascular Research Panel (10,430 PCR amplicons covering 404 genes known to harbor mutations affecting cardiovascular function). Variants with low quality score less than 30 were excluded. Synonymous mutations without amino acid changes were excluded. Variants were classified to be benign when present in Human Genetic Variation Database (HGVD)[39] or ESP 6500[40] database with an allele frequency more than 1%. After filtering, we identified the heterozygous 3 base pairs deletion (c.478_480del, p.Δ160E) in *TNNT2*, 1-base pair missense mutation (c.C78325T, p.R26109C) in *TTN*, which encodes titin, 1-base pair missense mutation (c.G947T, p.R316L) in *RYR1*, which encodes ryanodine receptor in skeletal muscle, and 1-base pair missense mutation (c.G3134T, p.R1045L) in *PTPN14*, which encodes a member of the protein tyrosine phosphatase family, in the proband. Among these four variants, Δ160E in *TNNT2*, R26109C in *TTN* and R1045L in *PTPN14* were commonly identified in the proband and his three daughters with HCM. Δ160E in *TNNT2* is defined as a pathogenic variant in HCM cohorts,[20] whereas either *TTN* or *PTPN14* has not been reported as a causative gene for HCM.

Cell culture, generation of human iPSCs and differentiation to cardiomyocytes

HEK293T cells were maintained in high glucose Dulbecco’s Modified Eagle Medium (DMEM, Gibco) containing 10% fetal bovine serum (FBS, Sigma-Aldrich) and penicillin, streptomycin and glutamine (PSG, Gibco). iPSCs were generated from peripheral blood mononuclear cells (PBMCs) obtained from the proband harboring the heterozygous *TNNT2* Δ160E mutation. PBMCs were separated from peripheral whole blood using Ficoll-Plaque (GE). Reprogramming was performed using Sendai virus vectors with OCT3/4, SOX2, KLF4, and c-MYC (CytoTune-iPS 2.0 Sendai Reprogramming Kit, Life Technologies). iPSCs were cultured under feeder-free conditions using StemFit AK02N (AJINOMOTO) as described previously[41] on a laminin-coated plate. iPSCs were differentiated into cardiomyocytes using a chemically defined protocol as described previously.[15] The culture medium was replaced with RPMI 1640 medium (ThermoFisher Scientific, USA) with recombinant human albumin (Sigma-Aldrich) and L-ascorbic acid 2-phosphate (Sigma-Aldrich) for differentiation. iPSCs were treated with CHIR99021 (LC Laboratories, USA) (day 0–2), Wnt-C59 (Selleck Chemicals, USA) and XAV-939 (Cayman) (day 2–4). On day 11, iPSC-CMs were dissociated with 0.25% Trypsin-EDTA (Gibco, USA) and replated into a 6-well plate (IWAKI) precoated with gelatin (Nitta Gelatin) and incubated with DMEM containing 10% FBS, 1% Penicillin/Streptomycin and 2 mmol/L L-Glutamine (PSG, Gibco, Thermo Fisher Scientific). We then initiated electrical stimulation at 2 Hz, with an electric field of 0.5 Volt/mm, and with 5 ms pulses (C-Pace EM, IonOptix) and continued it for 1 week. After that iPSC-CMs were replated into gelatin-coated 24-well plates or 96-well μClear plates (Greiner) and incubated with DMEM containing serum for further analysis.

Immunofluorescent staining

iPSCs were seeded into 96-well μClear plates (Greiner) at the density of 1,000 cells/well and incubated at 37ºC for colony formation. iPSC-CMs were treated with 0.25% Trypsin-EDTA, suspended with DMEM containing 10% FBS, PSG and 10 μmol/L Y-27632 (Wako) and filtered with a 100 μm cell strainer (FALCON). iPSC-CMs were replated into 96-well μClear plates precoated with gelatin (Nitta Gelatin) at the density of 10,000 to 20,000 cells/well. For immunostaining, cells were fixed with 4% paraformaldehyde for 15 min, permeabilized with 0.5% Triton X-100 for 20 min and blocked with 1% bovine serum albumin (BSA) in PBS for 30 min at room temperature. Cells were incubated with primary antibodies diluted by 1% BSA for overnight at 4°C, and with secondary antibodies conjugated with Alexa Fluor Dyes (Molecular Probe) including Hoechst 33342 to stain the nuclei for 45 min at room temperature. For tissue immunofluorescent staining, myocardium samples were embedded in Tissue-Tek OCT cryo-embedding compound (Miles Laboratories). Cryostat sections at 5 μm were fixed in acetone for 10 min, rinsed 3 times in PBS and blocked with 1% BSA in PBS for 30 min. Samples were then incubated with primary antibodies for overnight at 4°C. After washing with PBS, samples were stained with appropriate secondary antibodies for 1 h, and Nuclei were stained with Hoechst 33342. Stained slides were rinsed with PBS and mounted in a FluorSave Reagent (Merck Millipore). Immunofluorescent images were acquired using fluorescence microscope BZ-X700 (Keyence) and a spinning disk-based confocal microscope (IXplore SpinSR; Olympus). For high-content imaging analysis, immunostaining images from iPSC-CMs in 96-well plates were obtained using IN Cell Analyzer 6000 (GE healthcare) and the quantitative analyses were performed using IN Cell Developer Toolbox (GE) as described previously.[25,26] The ability to differentiate into the three germ layers was verified using the Human Pluripotent Stem Cell Functional Identification Kit (R&D Systems).

Cel-I assay

HEK293T cells were seeded in a 24-well plate (5×10^4 cells/well) and a pX459 vector was transfected into HEK293T cells using Lipofectamine 3000 (Life Technologies) on the day after seeding. Two days after transfection, medium was replaced to the medium containing 2.0 μg/mL puromycin to select the cell transfected with a pX459 vector. After puromycin selection, genomic DNA was extracted using QIAamp DNA Mini Kit (QIAGEN). Target regions were amplified by PCR (KOD Fx Neo, TOYOBO) as follows: 94°C for 2 min, followed by 30 cycles of 98°C for 10 s, annealing temperature (depending on primer sequences) for 30 s and 68°C for 40 s. Primer sequences are listed in Supplementary Table. PCR products were purified using QIAquick PCR purification kit (QIAGEN) and PCR fragments both from untreated and treated allele were hybridized to form hetero DNA duplex. The hybridized PCR hetero duplexes were then enzymatically digested by mismatch-specific endonuclease, Cel-I 42°C for 60 min (SURVEYOR Mutation Detection Kit) and electrophoresed.

Plasmid construction

The gRNA sequences targeting the genomic region surrounding 478_480 deletion mutation in *TNNT2* were designed using CRISPR Design Tool,[42] and cloned into a pX459 vector (Addgene plasmid # 62988) as described previously.[43] DNA sequences for 5'-terminal and 3'-terminal homology arms surrounding 478_480 deletion mutation in *TNNT2* gene were amplified from genomic DNA of the proband, and then cloned into pCR bluntII-TOPO vector (Thermo). Full length human *TNNT2* coding sequence was amplified from complementary DNA of the proband and cloned into pENTR/D-TOPO vector (Thermo). For adeno-associated virus (AAV) generation, full length *TNNT2* coding sequence was subcloned into a pAAV vector (TaKaRa). The red fluorescent genetically-encoded calcium indicators for optical imaging (RGECO) sequence was amplified from CMV-R-GECO vector[44] (Addgene plasmid # 32444) and inserted into 5' terminus of the *TNNT2* coding sequence in a pAAV vector.

Generation and purification of AAV

To generate AAV2, HEK293T cells were transfected with a pAAV vector encoding RGECO-*TNNT2* (WT and Δ160E), a pHelper vector and a pRC2-mi342 vector (AAVpro Helper Free System, TaKaRa) using calcium phosphate transfection (CalPhos Mammalian Transfection Kit, TaKaRa). Seventy-two h after transfection, HEK293T cells were detached by addition of 1/80 volume of 0.5 mol/L EDTA (pH 8.0), then pelleted via low-speed centrifugation (2000 × g for 10 min). Cell pellet was lysed with AAV Extraction Solution A and centrifuged (9000 × g for 10 min). AAV Extraction Solution B was added to the obtained supernatant and stored at -80°C. Collected AAV was purified using AAVpro Purification Kit (TaKaRa), and viral titer was calculated using AAV Titration Kit (TaKaRa).

RNA extraction, quantitative real-time PCR

Total RNA was extracted using RNeasy mini kit (QIAGEN) and converted to cDNA using high capacity RNA-to cDNA RT kit (Thermo). Quantitative real-time PCR was performed using SYBR green or probe method (THUNDERBIRD SYBR, probe qPCR mix, TOYOBO). All samples were processed in duplicate. The each transcript was quantified by the threshold cycle (Ct) method using GAPDH as internal control. PCR primers and probes used for quantitative PCR are listed in Supplementary Table.

Droplet digital PCR and quantitative real-time PCR

Droplet digital PCR (ddPCR) was performed according to the protocol using the QX200 ddPCR system (Bio-Rad). To specifically detect transcripts from the WT allele or 478_480 deletion allele in *TNNT2*, HEX- or FAM-labeled probes were designed (assay ID: dMDS354511384, Bio-Rad). After PCR, the generated droplets were detected and analyzed

using a QX200 droplet reader (Bio-Rad).

Transfection of plasmids into human iPSCs and selection of targeted clones.

Plasmid constructs for genome editing were transfected into iPSCs as described previously[45,46] with some modifications. Five micrograms of pX459 plasmid were electroporated into 1×10^5 cells using a NEPA 21 electroporator (poring pulse: pulse voltage, 125 V; pulse width, 5 ms; pulse number, 2; Nepagene). For homology-directed repair (HDR)-mediated genome editing, 5 µg of repair template DNA plasmid (pCR bluntII-TOPO vector) was additionally transfected. Puromycin (0.2 µg/mL) was added on the day after electroporation. Twenty-four hours after puromycin addition, the culture medium was replaced with puromycin-free medium. Five days after transfection, iPSCs were passaged in 6-well plates at a density of 500 cells/well for clonal colony formation. Simultaneously, genomic DNA was extracted, and genome editing results were evaluated by direct sequencing. After iPSC colony formation, each colony (at least 24 colonies) was picked up and dissociated into single cells in a sterile tube. The cell suspensions were split into two 96-well plates for genotyping and cell expansion. Genomic DNA was then extracted, the target genomic region was amplified by PCR, and then evaluated by direct sequencing or sequence analysis after cloning into pCR bluntII-TOPO vectors. To obtain targeted single clone iPSCs, cells were subjected to repeated passages into a new culture dish for clonal colony formation.

Myofilament-localized calcium dynamics

AAV encoding RGECO-*TNNT2* (WT, Δ160E, R92W and E163K) or *MYL2*-RGECO (WT and R58Q) was transduced into iPSC-CMs with the WT *TNNT2* and *MYL2* gene. R92W mutation in *TNNT2* and R58Q mutation in *MYL2* are pathogenic variants which cause HCM. E163K in *TNNT2* is a likely pathogenic variant located at the adjacent position to Δ160E in troponin T.[20]

Five days after transduction, the fluorescence signals of RGECO-TnT or myosin light chain-RGECO fusion protein in iPSC-CMs were detected using an IXplore SpinSR (Olympus) equipped with a UPLANSAPO 20x/0.75 NA objective lens, a CSU-W1 scan unit (Yokogawa), and an ORCA-Flash 4.0 v3 CMOS camera (Hamamatsu). Laser excitation was provided at a wavelength of 561 nm and fluorescence emissions were acquired at wavelengths between 580.5 nm and 653.5 nm. Video imaging was obtained at a frame rate of 20 frames/s (fps) for 10 s.

Calcium transient measurements

Calcium transients in monolayered iPSC-CMs were assessed 7 days after replating at a density of 8×10^4 cells/well in a 96-well imaging plate (Greiner). iPSC-CMs were incubated in culture medium loaded with Cal-520 AM (4 μmol/L, AAT Bioquest Inc., Cat# 21130) for 20 min, followed by washout with culture medium and 30-min recovery before the experiments at 37°C. Whole-well fluorescence was measured and analyzed using FDSS/μCell (Hamamatsu Photonics) with a sampling frequency of 16 Hz, exposure time of 47 ms, excitation wavelength of 480 nm, emission wavelength of 540 nm, and temperature controlled at 37°C. The experimental plates were kept in the FDSS/μCell system for 10 min, and a baseline recording was performed for 2 min. To assess the effect of EGCG, the recording was repeated at 10 min after adding EGCG (100 μmol/L) as described previously[47] with some modifications. Each recording was performed using electric field stimulation at 1 Hz. The following parameters of calcium transients were measured: duration from the peak amplitude to 50% decay (decay time 50), duration from the bottom to the peak amplitude (time to peak), and duration with amplitude higher than half of the peak (peak width duration 50). For evaluation of calcium irregularities, we defined early increase in fluorescence intensity interspersed by regular calcium transient as irregular calcium transients as described previously.[48]

Motion vector analysis

The contractile properties of iPSC-derived cardiomyocytes were assessed using a Cell Motion Imaging System (SI8000, SONY) as described previously[49] with some modifications. Motion videos were recorded as sequential phase-contrast images using a 4× objective, at a frame rate of 150 fps, a resolution of 1024 × 1024 pixels, and a depth of 8 bits. The motion videos were obtained from at least four fields of four wells in each isogenic iPSC-CM cultured in a 24-well plate. To assess the effect of EGCG, we recorded motion videos in the same field at baseline and at 10 min after adding EGCG (100 μmol/L) in the set of isogenic iPSC-CMs. Data were acquired from at least three independent experiments. The maximum contraction velocity (CV), relaxation velocity (RV), contraction duration, relaxation duration, contraction-relaxation duration, contraction deformation distance, and relaxation deformation distance calculated as the area under the CV and RV were obtained.

Measurement of the electrical activity of the set of iPSC-CMs using MEA

The multielectrode array (MEA) recordings were performed using MEA data acquisition system (USB-ME64-System; Multi Channel Systems, Germany). The set of isogenic iPSC-CMs were dissociated at 4 weeks after differentiation and re-suspended in serum-supplemented culture medium with 40% high glucose DMEM (Sigma-Aldrich), 20% fetal bovine serum (FBS; Gibco, USA), 40% IMDM (Sigma-Aldrich), 1% minimum essential medium non-essential amino acid solution (Sigma-Aldrich), 0.5% L-glutamine (Sigma-Aldrich), and 0.1% penicillin-streptomycin (Gibco, USA). We recorded signals 7 days after the replating of iPSC-CMs on the MEA chips at a density of 1×10^6 cells/cm^2. Signals were recorded and analyzed using MC_Rack (Multi Channel Systems).[22,23] For quantitative evaluation of electrical activity, occurrence of abnormal electrical activity and field potential duration (FPD) was assessed during the one-minute observation in the set of isogenic iPSC-

CMs.

Western Blotting

Cells were washed with cold PBS and directly lysed with CHAPS Lysis buffer (1% CHAPS, 25 mmol/L Tris-HCl (pH7.4), 137 mmol/L NaCl, 2.68 mmol/L KCl, 5 mmol/L EDTA). The protein concentration was determined by BCA Protein Assay Kit (Thermo). Lysate samples were mixed with 4 × Laemmli sample buffer (Bio-Rad) with mercaptoethanol (2.5%). Proteins were separated by SDS-PAGE and transferred to PVDF membrane. After blocking with 3% nonfat milk for 1 h, the transferred membrane was incubated with primary antibody for overnight at 4°C and with secondary antibody for 45 min at room temperature. The immunoreaction enhancer solution (Can Get Signal, Toyobo) was used to detect calcium/calmodulin-dependent protein kinase IIδ (CaMKIIδ). The other antibodies were diluted by 3% nonfat milk. The membrane signals were detected by chemiluminescence using ECL or ECL prime reagent (GE). The protein expression level was quantified using ImageQuant TL (GE). The expression level of each protein was normalized by that of GAPDH.

Statistical Analysis

Unless otherwise noted, data are represented as medians and interquartile ranges from at least three independent experiments. Statistical analysis was performed using JMP® 14.3.0 (SAS Institute Inc., Cary, NC, USA). Graphs were made with GraphPad Prism v9.1.0 (GraphPad Software, Inc., La Jolla, CA, USA). The data were analyzed by the Shapiro-Wilk test to test for normal distribution. Student's *t*-test or ANOVA were used to compare differences between continuous variables that were normally distributed. The Tukey-Kramer test was used for post-hoc analysis (Figure 5D). If the data were not normally distributed, the Mann-Whitney (Figure

2A, 3F) and Kruskal-Wallis rank sum tests were used (Figure 1E, 2F, 2I, 3B, 4B, 4E, 5B, 6B and 6C). The Steel-Dwass test was used for post-hoc analysis. Paired data were compared using the Wilcoxon signed-rank test (Figure 7A and 7B). Statistical significance was set at $p < 0.05$.

Supplemental Table I

Information of PCR primers and oligo DNA nucleotides used in this manuscript were listed.

Oligo DNA		sequence (5'-3')	Application
TNNT2_gRNA	sgRNA	GGAAGAGAGGGCTCGACGAG	
Hs_TNNT2_mRNA_F	primer	CACCatgtctgacatagaagaggt	cloning for pENTR vector to generate full length Hs TNNT2 CDS
Hs_TNNT2_mRNA_R	primer	ctatttccagcgcccggt	cloning for pENTR vector to generate full length Hs TNNT2 CDS
Hs_TNNT2_F_2	Fw	aggacctcattcaatcacgcag	primer for detection of genomic cleavage at TNNT2 locus
Hs_TNNT2_R_2	Rev	ggcctgctaaaccgggaaacca	primer for detection of genomic cleavage at TNNT2 locus
Hs_TNNT2_synonymous_F	primer	GAGGGCTCGAAGAGAAGAGG	primer for inverse PCR to generate HDR template with synonymous mutation
Hs_TNNT2_synonymous_R	primer	TCTTCctgatttacagcagg	primer for inverse PCR to generate HDR template with synonymous mutation

Supplemental Figure I

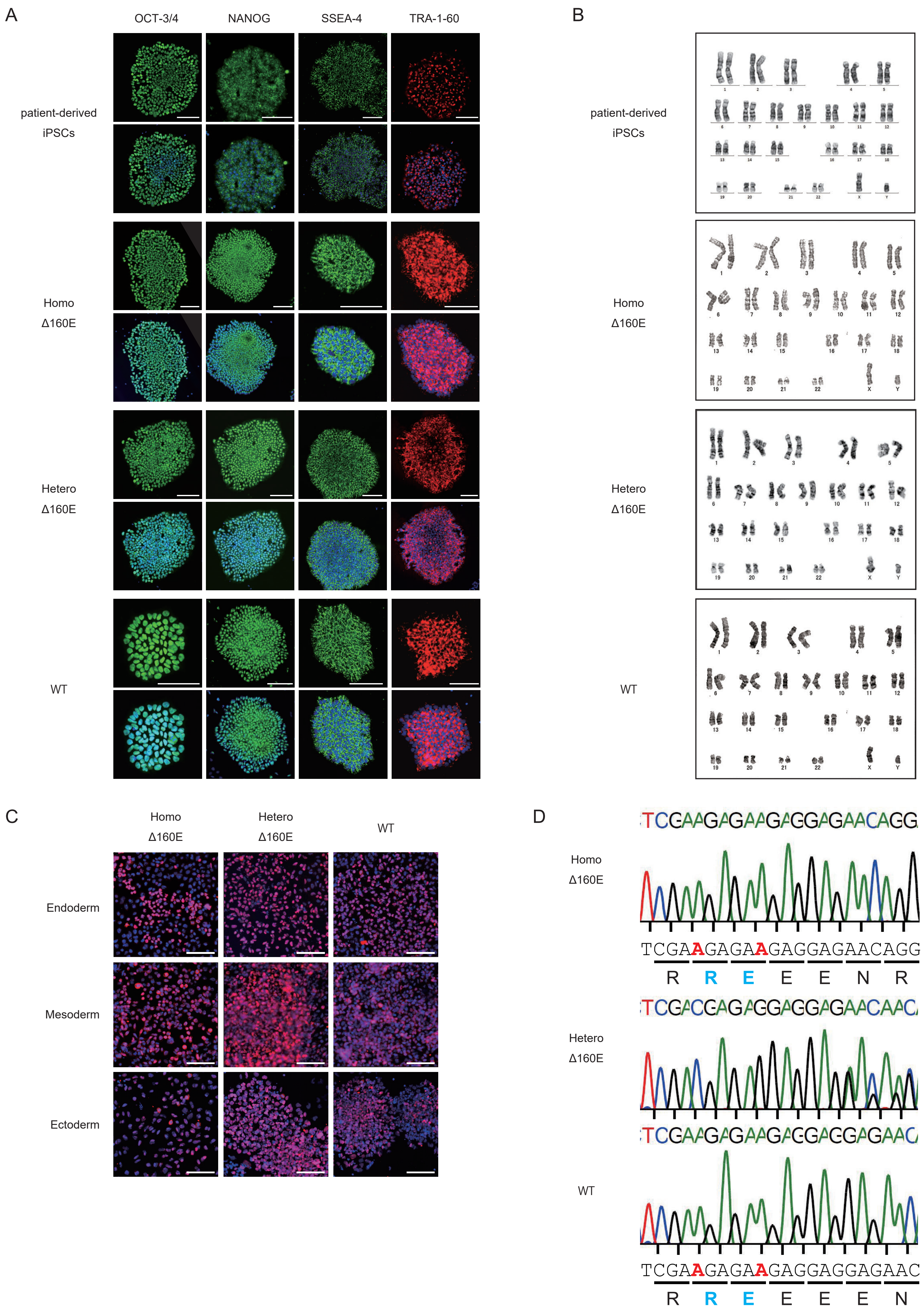

Supplemental Figure I

A) The patient-derived iPSCs and the generated set of isogenic iPSCs were fixed and immunostained with the indicated pluripotent markers. Nuclei were detected by Hoechst staining. Bar: 100 µm.

B) Karyotype analysis of the patient-derived iPSCs and the generated set of isogenic iPSCs.

C) The mesoderm, endoderm, and ectoderm derivatives from the generated set of isogenic iPSCs were fixed and immunostained with the markers of three germ layers, BRACHYURY, SOX17, and OTX2. Nuclei were detected by Hoechst staining. Bar: 100 µm.

D) Direct Sanger sequence analysis of TNNT2 locus using genomic DNA obtained from the generated isogenic iPS clones. In Homo-Δ160E and WT clone, inserted synonymous mutations were indicated in red bold type.

Supplemental Figure II

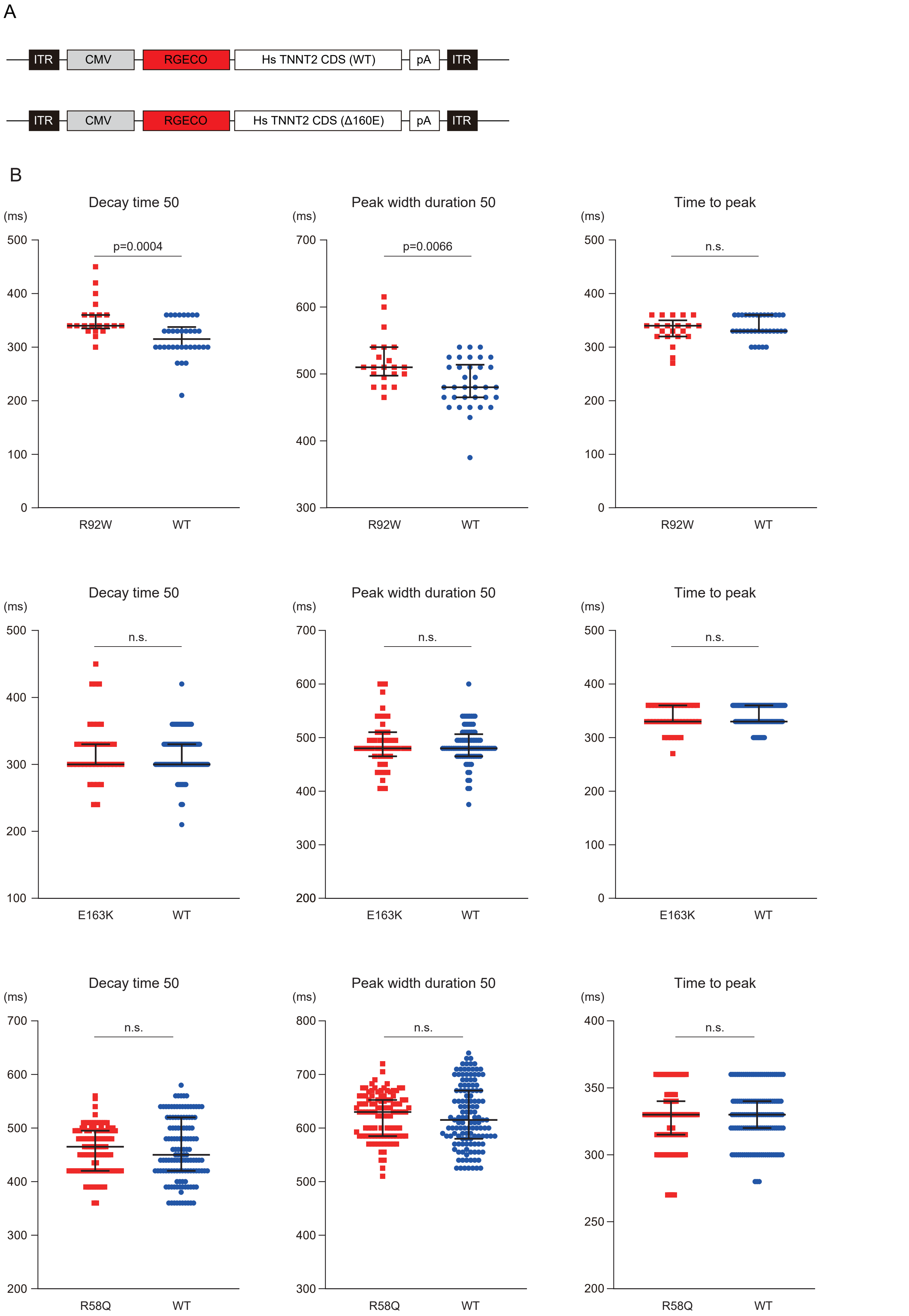

Supplemental Figure II

A) 5'-terminally RGECO-conjugated full length human TNNT2 coding sequence (Hs TNNT2 CDS, WT and Δ160E) with NotI linker was subcloned into the expression vector with CMV promoter and poly A sequence (pA) to generate AAV2. ITR: inverted terminal repeats.

B) The extracted parameters were analyzed in iPSC-CMs expressing RGECO-TnT (R92W, n = 21 cells) and RGECO-TnT (WT, n = 34 cells). Each data point is shown as a Dot plot for both groups. R92W mutation in TNNT2 prolonged the decay time 50 (340 [335-360] ms in R92W and 315 [300-338] ms in WT) and peak width duration 50 (510 [498-540] ms in R92W and 480 [465-514] ms in WT) of the myofilament-localized calcium transients in iPSC-CMs, while there was no significant difference in time to peak (340 [320-350] ms in R92W and 330 [330-360] ms in WT). The same analysis was performed in iPSC-CMs expressing RGECO-TnT (E163K, n = 40 cells) and RGECO-TnT (WT, n = 92 cells). E163K mutation did not have an influence on the decay time 50 (300 [300-330] ms in E163K and 300 [300-330] ms in WT), peak width duration 50 (480 [465-510] ms in E163K and 480 [465-506] ms in WT), and time to peak (330 [330-360] ms in E163K and 330 [330-360] ms in WT) of the myofilament-localized calcium transients in iPSCCMs. The same analysis was performed in iPSC-CMs expressing myosin light chain-RGECO (R58Q, n = 108 cells and WT, n = 129 cells). R58Q mutation in MYL2 did not have an impact on the decay time 50 (465 [420-495] ms in R58Q and 450 [420-520] ms in WT), peak width duration 50 (630 [585-653] ms in R58Q and 615 [580-670] ms in WT), and time to peak (330 [315-340] ms in R58Q and 330 [320-340] ms in WT) of the myofilament-localized calcium transients in iPSC-CMs.

A

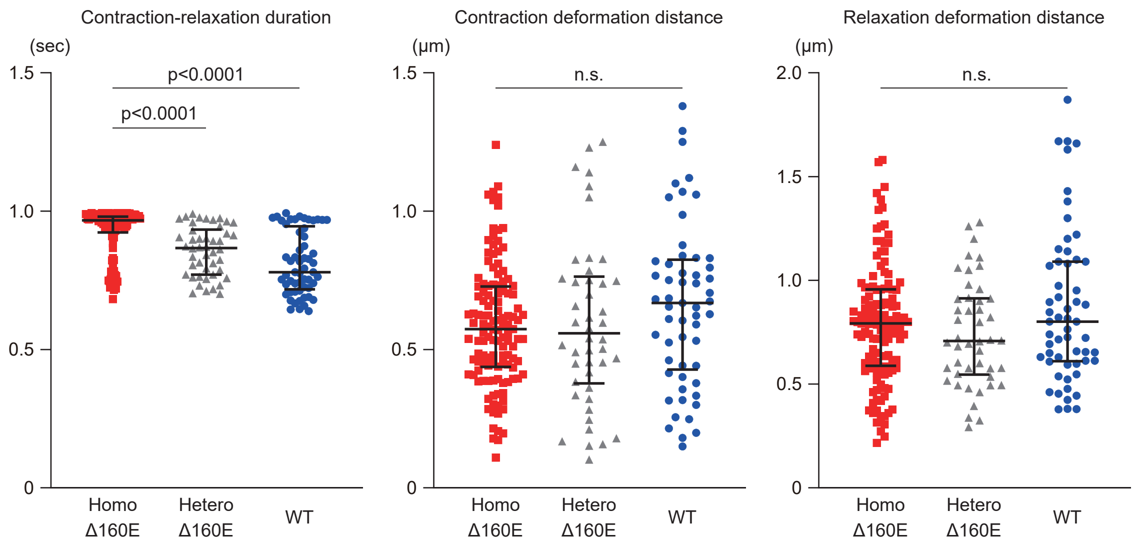
Contraction-relaxation duration
(sec)
1.5
1.0
0.5
0
p<0.0001
p<0.0001
Homo Δ160E
Hetero Δ160E
WT
Contraction deformation distance
(μm)
1.5
1.0
0.5
0
n.s.
Homo Δ160E
Hetero Δ160E
WT
Relaxation deformation distance
(μm)
2.0
1.5
1.0
0.5
0
n.s.
Homo Δ160E
Hetero Δ160E
WT

B

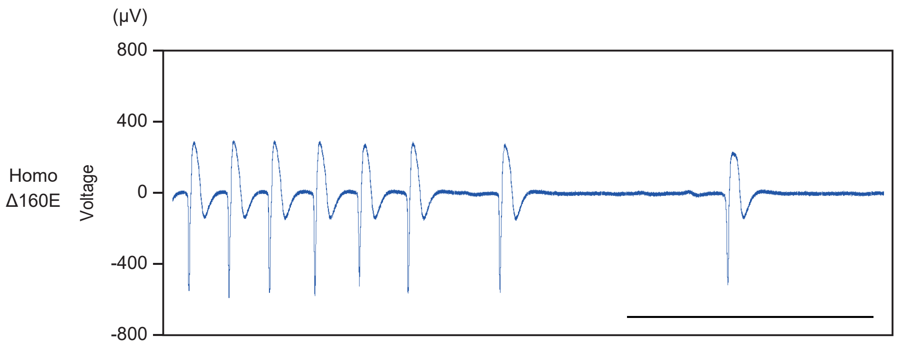
(μV)
800
400
0
-400
-800
Homo Δ160E
Voltage

Supplemental Figure III

A) In contractile properties, Homo-Δ160E-iPSC-CMs had prolonged contraction-relaxation duration (967 [924-980] ms) than the other isogenic iPSC-CMs (867 [771-934] ms in Hetero-Δ160E-iPSC-CMs, $p<0.0001$ and 779 [718-946] ms in WT-iPSC-CMs, $p<0.0001$). There was no significant difference in contraction deformation distance and relaxation deformation distance of the set of isogenic iPSC-CMs. Data was shown as a Dot plot in each group.

B) Representative waveform of abnormal electrical activity recorded by MEA in Homo-Δ160E-iPSC-CMs. Scale bar: 10 s. The abnormal electrical activity was observed only in Homo-Δ160E-iPSC-CMs (n = 1/9 analyzed samples in Homo-Δ160E-iPSC-CMs, n = 0/9 in Hetero-Δ160E-iPSC-CMs, n = 0/7 in WT-iPSC-CMs).
